# Attenuation of LPS-induced acute lung injury by continentalic acid in rodents through inhibition of inflammatory mediators correlates with increased Nrf2 protein expression

**DOI:** 10.1186/s40360-020-00458-7

**Published:** 2020-11-25

**Authors:** Hassan Ali, Ashrafullah Khan, Jawad Ali, Hadayat Ullah, Adnan Khan, Hussain Ali, Nadeem Irshad, Salman Khan

**Affiliations:** Pharmacological Sciences Research Lab, Department of Pharmacy, Faculty of Biological Sciences, Quaid-i-Azam University, Islamabad, Pakistan

**Keywords:** Continentalic acid, LPS, Inflammation, Cytokines, Antioxidants

## Abstract

**Background:**

Acute lung injury (ALI) together with acute respiratory distress syndrome (ARDS) are associated with high rate of mortality and morbidity in patients. In the current study, the anti-inflammatory effects of continentalic acid (CNT) in LPS-induced acute lung injury model was explored.

**Methods:**

The acute lung injury model was established by administering LPS (5 mg/kg) intraperitonealy. Following LPS administration, the survival rate, temperature changes and lung Wet/Dry ratio were assessed. The antioxidants (GSH, GST, Catalase and SOD) and oxidative stress markers (MDA, NO, MPO) were evaluated in all the treated groups. Similarly, the cytokines such as IL-1β, IL-6 and TNF-α were analyzed using ELISA assay. The histological changes were determined using H and E staining, while Nrf2 and iNOS level were determined using immunohistochemistry analysis. The molecular docking analysis was performed to assess the pharmacokinetics parameters and interaction of the CNT with various protein targets.

**Results:**

The results showed that CNT dose dependently (10, 50 and 100 mg/kg) reduced mortality rate, body temperature and lungs Wet/Dry ratio. CNT post-treatment significantly inhibited LPS-induced production of pro-inflammatory cytokines such as IL-1β, IL-6 and TNF-α. The CNT post-treatment markedly improved the hematological parameters, while significantly reduced the MPO (indicator of the neutrophilic infiltration) activity compared to the LPS treated group. Furthermore, the CNT (100 mg/kg) post-administration remarkably inhibited the lung Wet/Dry ratio. The CNT (100 mg/kg) treated group showed marked reduction in the oxidative stress markers such as malonaldehyde (MDA) and Nitric oxide (NO) concentration, while induced the level of the anti-oxidant enzymes such as GST, GSH, Catalase and SOD. Similarly, the CNT markedly reduced the iNOS expression level, while induced the Nrf2 protein expression. Additionally, the molecular docking study showed significant binding interaction with the Nrf2, p65, Keap1, HO-1, IL-1β, IL-6, TNF-α and COX-2, while exhibited excellent physicochemical properties.

**Conclusion:**

The CNT showed marked protection against the LPS-induced lung injury and improved the behavioral, biochemical and histological parameters. Furthermore, the CNT showed significant interaction with several protein targets and exhibited better physicochemical properties.

## Background

Acute lung injury (ALI) together with acute respiratory distress syndrome (ARDS) are respiratory syndromes associated with high mortality and morbidity caused by pneumonia, trauma and sepsis [1]. ALI is an inflammatory disease of lungs which is characterized by the disruption of alveolar endothelial and epithelial barriers, neutrophilic infiltration at pulmonary sites together with non-cardiogenic edema [2, 3]. Despite improvements in therapies, the morbidity and mortality rates of ALI remain high i.e. 30–40% [4–6]. Thus, novel therapies with proven safety and efficacy are needed to improve the clinical outcomes of the patients affected with the disease. LPS is a glycolipid that is present in the Gram-negative microbes cell wall, is considered as the core basis of ALI [7]. LPS induces ALI in animal models by altering alveolar membrane permeability, recruiting activated neutrophils and macrophages to the lungs [8, 9]. These effects compromises the alveolar membrane integrity and ultimately impairs the gaseous exchange [8, 9]. Furthermore, LPS exposure is associated with exaggerated production of various pro-inflammatory cytokines such as tumor necrosis factor (TNF)-α, interleukin (IL)-1β and interleukin (IL)-6 in lungs [8]. Over production of TNF-α, IL-1β and IL-6 lead to the development of ALI and results in poor clinical outcome in patients with ALI [10, 11].

The Nrf2 signaling plays an important role in neutralizing the oxidative stress and oxidative stress mediated damage [12]. The Nrf2 remains dormant within the cytosol under the inhibitory influence of the Keap1 and translocated to nucleus when become free from the Keap1 influence. Following translocation to nucleus, the Nrf2 interact with the antioxidant response element (ARE) to alter the expression of the genes concerned with the antioxidants and cytokines [13, 14]. Previously reported studies suggest that over production of inflammatory cytokines and oxidative stress triggered by LPS leads to neutrophil infiltration in the lung tissue [15]. Persistent activation and migration of neutrophils into the transepithelial membrane is considered as a hallmark feature of acute lung injury that increases the destruction of basement membrane with promotion of membrane permeability [16]. Significant evidence reported the involvement of inflammatory response and oxidative stress in the pathogenesis of ALI [17]. The results observed include decreased level of antioxidant enzymes (GSH, GST, SOD and CAT), abundant production of neutrophils and elevated levels of inflammatory cytokines (IL-1β, TNF-α and IL-6) in plasma and lung tissue of mice [8]. Elevated plasma levels of cytokines i.e. IL-1β, IL-6 and tumor necrosis factor-α (TNF-α) strongly predicts the high mortality associated with acute lung injury [10, 18, 19].

Currently, numerous studies revealed that bioactive compounds from natural herbs act as potential candidates for the management of ALI in rodent model [20]. Continentalic acid is a diterpene obtained from *Aralia continentalis* which belongs to the family of Araliaceae [21]. Studies have been reported that continentalic acid exhibit numerous pharmacological activities such as anti-inflammatory, antiarthritic, nephroprotective etc. [22]. Therefore, in the present study, LPS-induced lung injury model in mice was used to simulate acute lung injury and observed the effects through the behavioral and biochemical methods. It was hypothesized that continentalic acid possesses its anti-inflammatory effect via inhibiting inflammatory response and oxidative stress in LPS-induced lung injury model.

## Methods

### Chemical and reagents

Continentalic acid (purity 99.9%) was received from Prof. Yeong Shik Kim, Emiritus Professor, College of Pharmacy, Seoul National University, Korea. All the chemicals and reagent included in this study such as dexamethasone and LPS were obtained from the Sigma Aldrich (St. Louis, MO, USA). The cytokines were analyzed in lung tissue using ELISA Kits obtained from the eBioscience (eBioscience, Inc. USA). The primary and secondary antibodies for Nrf2 and iNOS were obtained from the Santa Cruz (Santa Cruz Biotechnology, Inc). The DAB reagent was obtained from the Sigma Aldrich (St. Louis, MO, USA). The NGS (normal goat serum) and AB complex used in the immunohistochemistry were obtained from (SCBT, U.S.A).

### Animals and ethical statement

Male albino mice (BALB/c) (22–26 g) were used for the entire study having age of 3 to 4 weeks and purchased from National Institute of Health (NIH) Islamabad, Pakistan. Standard environmental and food conditions were provided to all the animals i.e. 22 ± 1 °C, 55 ± 5% humidity and 12 h light/dark cycle with free food and water access. All animal experimentations were carried out as per Bioethical Committee protocols for laboratory Animals Care and Use (Quaid-i-Azam University, Islamabad) under Ethical Committee code (Approval No. BEC-FBS-QAU 2018–86).

### Model and grouping

All the animals were randomly and double blindly assigned to six groups to avoid the experimental biasness (each group contain 8 mice). The continentalic acid and dexamethasone was dissolved in the normal saline (2% DMSO), while the LPS was dissolved in the normal saline only.

1. Vehicle control treated with normal saline (i.p)
2. Negative control treated with LPS 5 mg/kg only (i.p)
3. LPS (5 mg/kg, i.p) + vehicle
4. Positive control treated with the LPS + dexamethasone (10 mg/kg, i.p)
5. Continentalic acid (10 mg/kg, i.p) + LPS 5 mg/kg (i.p)
6. Continentalic acid (50 mg/kg, i.p) + LPS 5 mg/kg (i.p)
7. Continentalic acid (100 mg/kg, i.p) + LPS 5 mg/kg (i.p)

### In-vivo model of ALI and sampling protocols

To assess the percent survival rate during entire study, LPS 5 mg/kg with different doses of continentalic acid (10, 50 and 100 mg/kg body weight dissolved in normal saline (2% DMSO)) was administered intraperitoneally thirty minutes prior to LPS injection. The mortality of mice was recorded every 3 h after the LPS injection in each group for 24 h. At the end of the experiment, the animals were anesthetized with the combination of Xylazine and Ketamine injection (16 mg and 60 mg respectively, i.p) to make them unconscious and lessen the painful feeling related with the euthanasia. Once the animals were anesthetized, the CO_2_ chamber was used to euthanize the animals. The animal death was confirmed by assessing the heartbeat, respiration, eye reflexes and body movement. The overall euthanasia process was regulated by the institutional ethical committee.

### Temperature assessment

LPS is the endotoxin of the Gram negative bacteria and is associated with increase in the body temperature [23]. The pyrexia was measured before and after LPS induction at 0 and 24 h according to the previously described method [24].

### Determination of lung wet/dry weight ratio

In order to assess the pulmonary edema following LPS administration, the wet/dry weight ratio was determined [25]. Mice were sacrificed 24 h after LPS stimulation. Wet weight was determined after excising lung tissues. The wet lung was then placed in the oven at 80 °C for 24 h to measure the dried weight. Then W/D weight ratio was calculated by dividing dry weight over wet weight [25].

### Determination of GSH, GST, catalase and SOD concentrations

The tissue GSH, GST, Catalase and SOD levels were determined according to the previously established protocols [14]. Briefly, lung tissues were homogenized and spun at 448 RCF (relative centrifugal force) for 10 min. The supernatant obtained was then used for the determination of enzymatic and non-enzymatic antioxidant activity. The concentrations of GSH, GST, Catalase and SOD were detected by monitoring the change in absorbance using spectrophotometer at λmax 412 nm, λmax 340 nm, λmax 240 nm and λmax 413 nm, respectively [14].

### Estimation of lipid peroxidation

MDA level was determined according to previously reported method [26]. Briefly lung tissues were first homogenized and then centrifuged at 448 RCF for 10 min. The supernatant obtained was then used for the determination of antioxidant activity. The presence of thiobarbituric acid reactive substances (TBARS) were detected by monitoring changes in absorbance using microplate reader at 535 nm [14].

### Determination of nitrite concentrations

The production of nitric oxide in all the treated groups were measured by Griess assay according to the method described previously [27]. At the final day of experiment, all the mice were sacrificed and blood collection was done through cardiac puncture. The collected blood was then centrifuged at 700 RCF for 10 min at 4 °C and plasma was separated from the cellular component for NO determination as reported previously [27].

### Cytokines analysis

Inflammatory cytokines (IL1-β, IL-6 and TNF-α) levels were determined in lung tissues using ELISA (enzyme linked immunosorbent assay) kits obtained from (eBioscience, Inc., USA). Cytokines level were determined as per manufacturer’s instruction [28, 29].

### Complete blood count

The immune cells plays key role during inflammatory conditions [30]. In order to assess the effect of continentalic acid on the various immune cells, blood complete count was performed. Following 24 h of LPS administration, the blood sample was collected from the heart of mice. The collected blood was then utilized in hematological studies by obtaining complete blood picture including major hematological parameters such as WBC, Red blood cells (RBC), platelets, Hemoglobin (Hb), neutrophils and lymphocytes [31].

### Tissue processing and sample collection

Mice were sacrificed 24 h after LPS administration using CO_2_ anesthetization and the entire lung tissue were dissected from all the groups. Separated lung tissue were washed by 0.9% normal saline and then preserved in 10% formalin for histopathological examination [32–34].

### Histopathological analysis

To illustrate the histological modifications induced by LPS, lungs were kept in formaldehyde solution and then fixed in PFA solution for 24 h. Subsequently, the tissues were sectioned at 5 μm and were stained with hematoxylin–eosin using standard histological techniques. The lung and tracheal tissues were examined under optical microscope, and photos were taken as described previously by [35, 36]. Results were graded from 0 to 4 for each item, as described above, where 0 = minimal damage, 1 = mild damage, 2 = moderate damage, 3 = severe damage and 4 = maximal damage as described previously [37].

### Myeloperoxidase assay for the neutrophilic infiltration

The MPO activity was performed to assess the inhibitory effect of the continentalic acid on the LPS-induced neutrophilic infiltration [38]. The MPO activity was determined using CTAB and o-dianisidine method as reported previously with necessary modification for all the treated group [38].

### Immunohistochemistry study

The immunohistochemistry was performed to determine the effect of the contenentalic acid on the Nrf2 and iNOS proteins [14]. The immunohistochemistry was performed as reported previously [14]. The tissue was deparrafinzed using xylene and washed in alcohol solution of different concentration. Following washing with alcohol the tissue were treated with the Avidin-biotin complex, followed by treated with NGS for 2 h, primary and secondary antibodies (Nrf2 and iNOS). At the end tissue were placed in the DAB solution, dried and cover slips were applied using mounting media. The slides were visualized at 100X microscope and quantified using Image J software 1.8_172 (NIH, USA) [14].

### Assessment of pharmacokinetic and pharmacodynamics analysis using docking studies

The pharmacokinetic parameters of the continentalic acid were assessed using Swiss target prediction software (http://www.swisstargetprediction.ch/) as reported previously [39]. The various parameters that were assessed during the current study includes physicochemical properties, lipophilicity, aqueous solubility, absorption and pharmacokinetic parameters were evaluated. The possible metabolic route and metabolites were also determined using Glory software (https://nerdd.zbh.uni-hamburg.de/glory/) [40]. Furthermore, docking analysis was performed to investigate the interaction with various molecular targets using AutoDock Vina program. The 3D structure of the continentalic acid using Chemdraw_16 and saved as PDB file. Furthermore, the toxicity of the continentalic acid was evaluated against the various cell lines using CLC-Pred (http://www.way2drug.com/Cell-line/) software. Similarly, the 3D-structures of target proteins (Keap-1 PDB-ID: 4iqk), (Nrf2 PDB-ID: 2flu), (p 65 PDB-ID: 1vkx), (HO-1 PDB-ID: 1ubb), (TNF-α PDB-ID: 2az5), (IL-1Β PDB-ID: 1itb), (IL-6 PDB-ID: 1p9m) and (COX-2 PDB-ID: 5ikq) were taken from RCSB protein data bank. Affinity of best docked pose of ligand and protein target complex was determined by E-value (Kcal/mol). The results of the docking interaction were analyzed using discovery studio visualizer_2016.

### Statistical analysis

Results were represented as the means (*n* = 8) ± Standard Deviations (S.D). The data was analyzed using One way analysis of variance (ANOVA) followed by Dunnett′s t test to compare means among groups. The “*p*” value less than 0.05 were considered statistically significant.

## Results

### Effects of continentalic acid on LPS-induced survival in mice

**Fig. 1:**
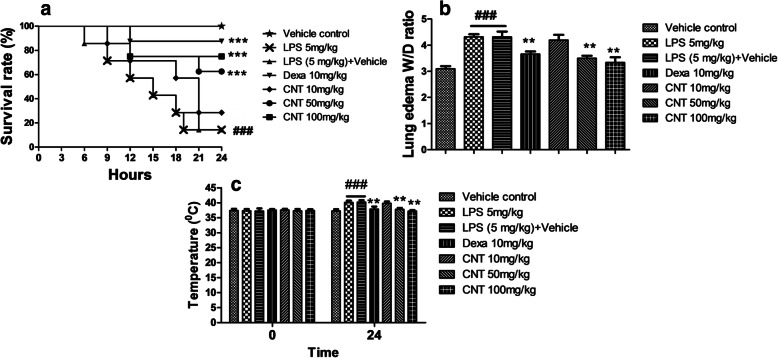
Effects of continentalic acid (10, 50 and 100 mg/kg) on LPS-induced mortality (**a**), lung W/D ratio (**b**) and temperature changes (**c**) in mice following LPS-induced lung injury. The survival rate was analysed at different time interval such as 0, 3, 6, 9, 12, 15, 18, 21 and 24 h following LPS-induced acute lung injury and the effect of the continentalic acid was assessed on the LPS-induced acute lung injury. The continentalic acid dose dependently improved the survival rate and the dose of the 100 mg/kg showed maximum protection. Similarly, the weight to dry ratio of the lung was assessed by dividing the wet weight of the lung on the dry weight. Furthermore, the temperature changes were determined in all the treated groups before the induction of the LPS-induced lung injury and 24 h after the LPS administration. The continentalic acid showed significant improvement in the survival rate, lung W/D ratio and temperature changes. All the data were expressed as mean (*n* = 8) ± SD. ^**###**^*p* < 0.001 compared to control group; ^*^p < 0.05, ^**^p < 0.01 and ^***^p < 0.001 compared to LPS-treated group

Treatment with continentalic acid significantly reduced mortality rate associated with LPS induction as shown in Fig. 1. The survival rate during 24 h in high dose of continentalic acid (100 mg/kg) treatment groups was significantly higher (80%) as compared to LPS groups (20%) (*p* < 0.01). However, continentalic acid at dose of 10 mg/kg did not have any protective effect in reducing mortality rate.

### Effects of continentalic acid on lung W/D ratio

The wet to dry ratio for all the treated groups were determined 24 h following LPS challenge. Significant increase in lung W/D weight ratio was observed for negative control groups as compared to normal. However, continentalic acid treatment significantly reduced lung W/D weight ratios dose dependently (*n* = 8, *p* < 0.001) as compared to LPS group. Similarly, the positive control treated with the dexamethasone also markedly reduced the wet to dry weight ratio compared to the negative control as shown in the Fig. 1.

### Effects of continentalic acid on LPS-induced pyrexia

The LPS (i.p) significantly increased the body temperature 24 h after the LPS administration. LPS group shown significant increase in body temperature after LPS administration. However, the dose of 50 and 100 mg/kg significantly reduced LPS-induced pyrexia dose dependently (*n* = 8, *p* < .001). Similarly, the positive control treated with the dexamethasone also significantly attenuated the body temperature as compared to the LPS group shown in the Fig. 1.

### Effect of continentalic acid on GSH, GST, catalase and SOD concentrations

**Fig. 2:**
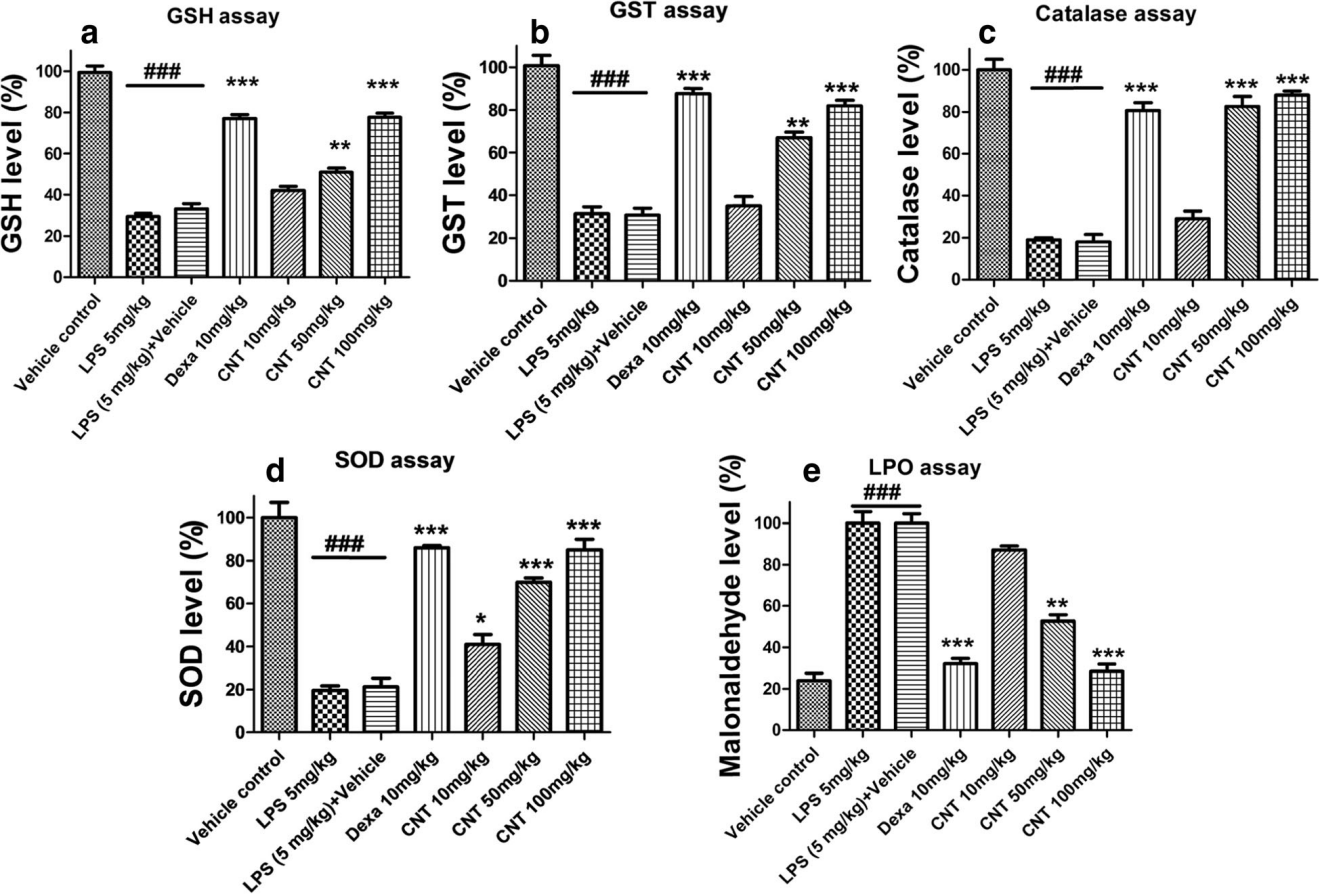
Effect of treatment with continentalic acid (10, 50 and 100 mg/kg) on levels of antioxidant enzymes such as (**a**) GSH, (**b**) GST, (**c**) Catalase, (**d**) SOD, (**e**) MDA in LPS-induced lung tissue. The level of these antioxidants and oxidative stress parameters were markedly compromised. However, continentalic acid post treatment significantly enhanced antioxidant enzymes such as GSH, GST, Catalase, SOD and reduced the level of MDA. The results were shown in percentage. All data were expressed as mean (*n* = 8) ± SD. ^**###**^*p* < 0.001 compared to control group; ^*^p < 0.05, ^**^p < 0.01 and ^***^p < 0.001 compared to LPS-treated group

LPS-induced oxidative stress was determined by measuring antioxidant enzymes (GSH, GST, CAT and SOD) in the lung tissue. It was noticed that LPS administration remarkably reduced GSH, GST, CAT and SOD levels as compared to control group. Continentalic acid treatment significantly elevated the GSH, GST, SOD and CAT levels (*p <* 0.01) as compared to negative control groups as evident from the Fig. 2.

### Effect of continentalic acid on lipid peroxidation (MDA level)

It was observed that LPS administration considerably enhanced malonaldehyde level in lung tissues as compared to normal group. Howerver, Continentalic acid remarkably reduced MDA level dose dependently as compared to LPS treated group (*p* < 0.001) as evident from the Fig. 2.

### Effect of continentalic acid on nitrite concentrations

**Fig. 3:**
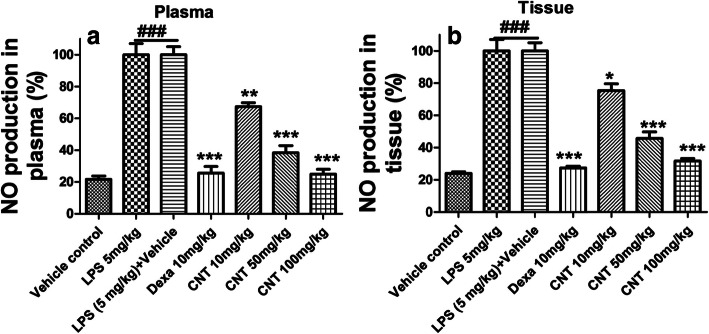
Effect of continentalic acid (10, 50 and 100 mg/kg) on LPS-induced nitrite concentration. The continentalic acid was assessed against the NO production in both plasma (**a**) and (**b**) tissue following establishing ALI. The continentalic acid treatment significantly reduced NO production in both plasma and tissue dose dependently. Similarly, the dexamethasone also markedly decreased the NO production in the both plasma and tissue. The results were shown in percentage. All data were expressed as mean (n = 8) ± SD. ^**###**^*p* < 0.001 compared to control group; ^*^p < 0.05, ^**^p < 0.01 and ^***^p < 0.001 compared to LPS-treated group

The nitrite level was significantly raised (*p* < 0.001) both in lung tissue and plasma post 24 h of LPS administration as compared to the control group. Continentalic acid remarkably reduced (*p* < 0.001) LPS-induced elevated nitrite level both in lung tissue and plasma. Similarly, dexamethasone also suppress nitrite level Fig. 3.

### Effect of continentalic acid on cytokine concentrations

**Fig. 4:**
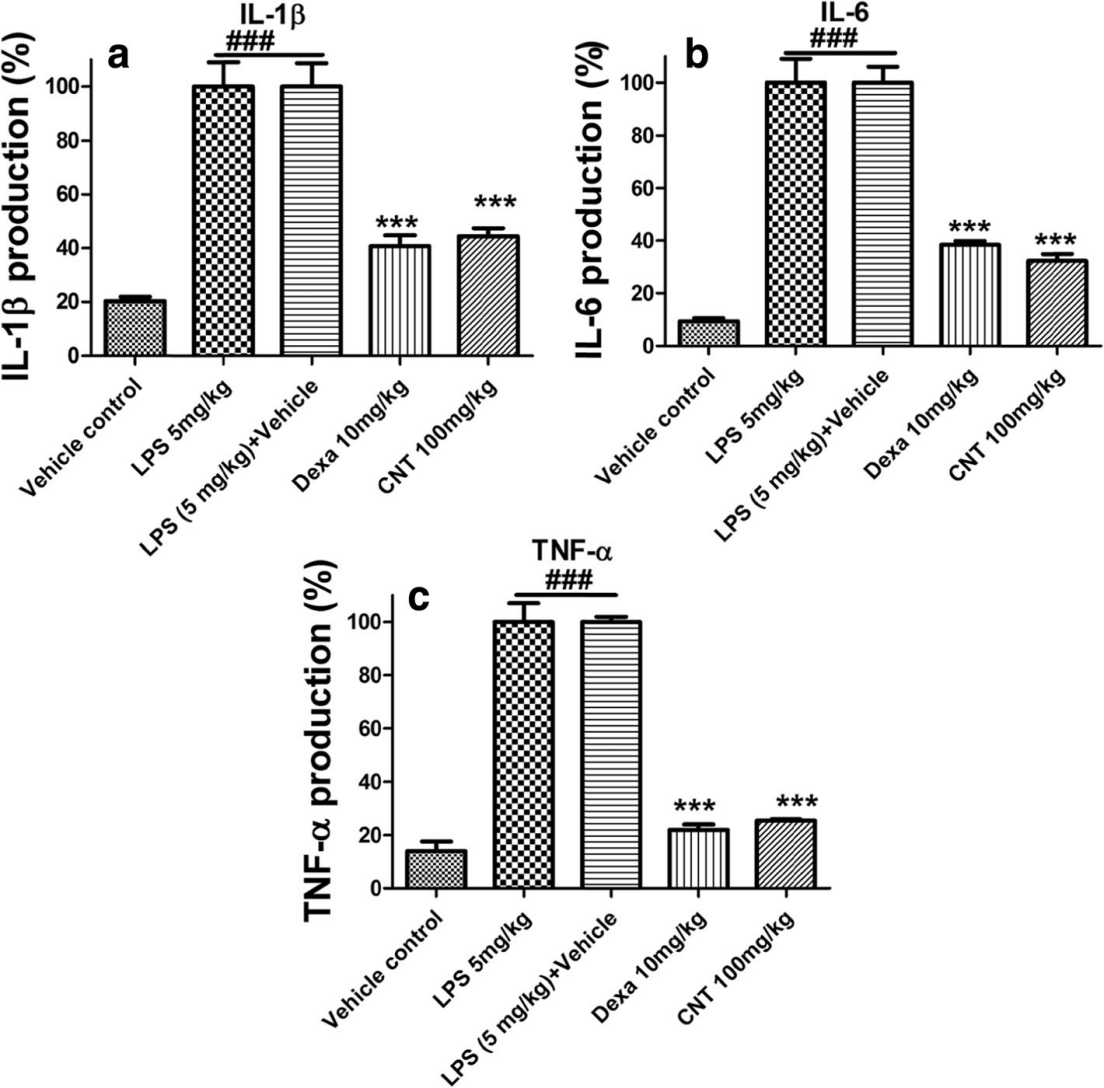
Effects of continentalic acid (10, 50 and 100 mg/kg) treatment on LPS-induced pro-inflammatory cytokines such as (**a**) IL-1β, (**b**) IL-6 and (**c**) TNF-α in lung tissue. The ELISA assay was performed to assess the cytokines production in all the treated groups following LPS-induced ALI. The LPS administration markedly increase the production of inflammatory cytokines in the negative control group, however, the continentalic acid treatment showed marked reduction in the inflammatory cytokines. The results of the assay were shown in the percentage. All data were expressed as mean (n = 8) ± SD. ^**###**^*p* < 0.001 compared to control group; ^*^p < 0.05, ^**^p < 0.01 and ^***^p < 0.001 compared to LPS-treated group

LPS-induced lung inflammation was determined by measuring pro-inflammatory cytokines (IL1-β, IL-6 and TNF-α) in lung tissue. LPS treated mice showed remarkable elevation of IL1-β, IL-6 and TNF-α level as compared to control group. However, continentalic acid treatment significantly inhibited production of pro-inflammatory cytokines, compared with LPS treated group (*p* < 0.001) Fig. 4.

### Effect of continentalic acid on CBC

As ALI is associated with neutrophilic infiltration into the lungs [15]. Complete blood count indicates severe neutrophilic infiltration in negative control groups vs vehicle control group as shown in Table 1. Treatment groups shown significant reduction in total neutrophils count in the lung tissue, especially continentalic acid at the dose of 50 mg/kg and 100 mg/kg showed maximum response.

**Table 1:** Effect of continentalic acid on CBC

Parameters	WBC count (10^**9**^/L)	LYM (10^**9**^/L)	NEU (10^**9**^/L)	RBC (10^**12**^/L)	PLT (10^**9**^/L)
**Vehicle control**	4.4 ± 0.17	3.6 ± 0.90	2.5 ± 0.12	4.62 ± 0.19	189 ± 10
**LPS treated**	8.5 ± 0.11^###^	6.9 ± 0.17^###^	6.5 ± 0.08 ^###^	4.41 ± 0.13^###^	185 ± 16^###^
**LPS + Vehicle**	8.34 ± 0.20^###^	7.12 ± 0.05^###^	6.7 ± 0.10 ^###^	4.32 ± 0.41^###^	184 ± 8^###^
**Dexa 10 mg/kg**	4.5 ± 0.21^***^	3.8 ± 0.11	2.60 ± 0.12^***^	4.49 ± 0.30	183 ± 10
**CNT 10 mg/kg**	6.5 ± 0.23	4.90 ± 0.09	5.01 ± 0.12	4.34 ± 0.11	189 ± 22
**CNT 50 mg/kg**	4.8 ± 0.15^**^	4.30 ± 0.12	2.70 ± 0.15^**^	4.51 ± 0.40	188 ± 13
**CNT 100 mg/kg**	4.4 ± 0.10^***^	3.70 ± 0.15	2.62 ± .90 ^***^	4.50 ± 0.09	182 ± 9

All data were expressed as mean (n = 8) ± SD

^**###**^*p* < 0.001compared to control group

^***^p < 0.001compared to negative control group

### Effect of continentalic acid on histopathology

**Fig. 5:**
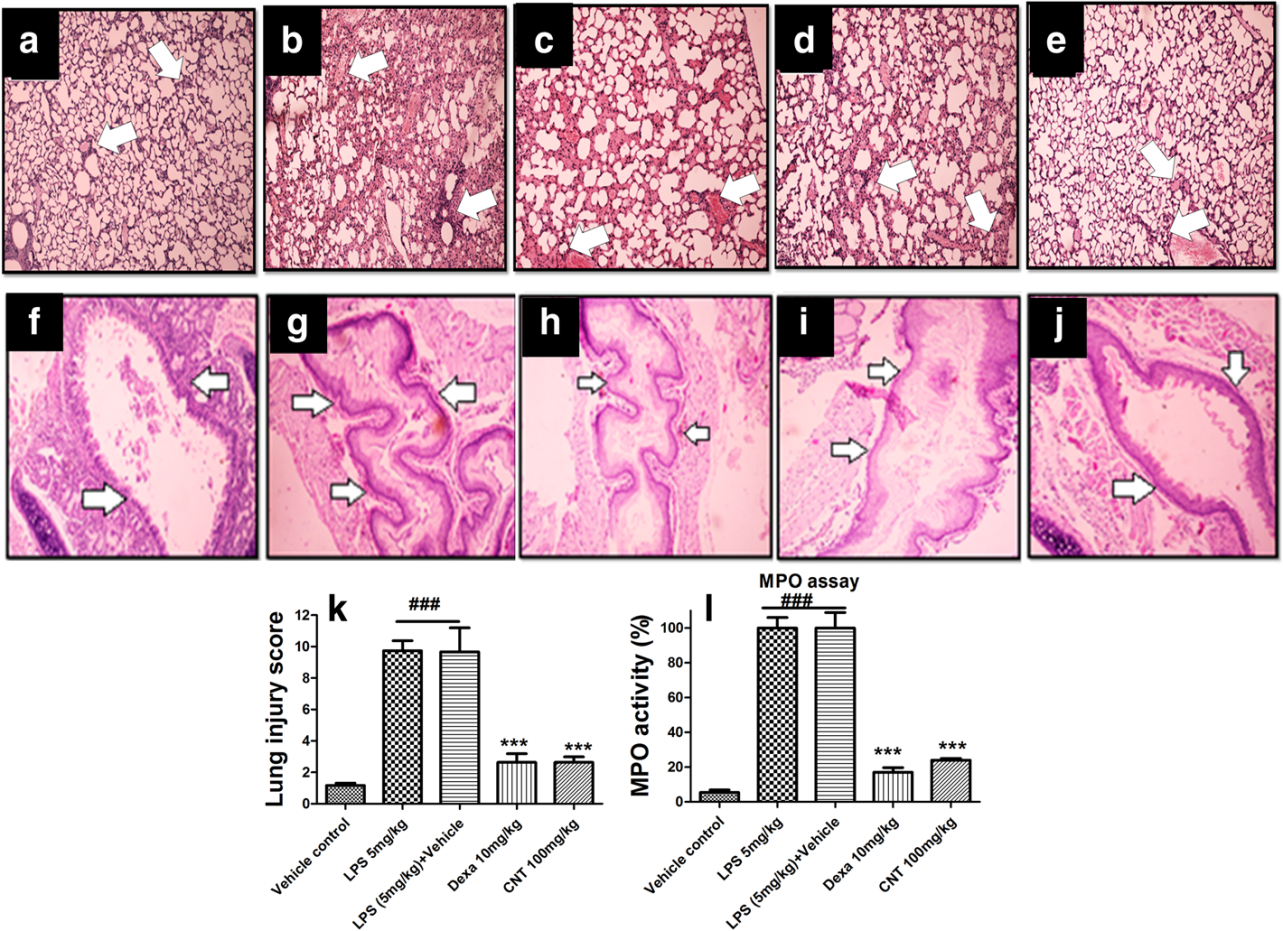
The effect of the continentalic acid (10 mg/kg, 50 mg/kg and 100 mg/kg) on the histopathological changes following LPS-induced lung injury using H and E staining in all the treated groups such as vehicle control (**a** and **f**), LPS 5 mg/kg (**b** and **g**), LPS (5 mg/kg) + Vehicle (**c** and **h**), dexamethasone (**d** and **i**) and CNT (**e** and **j**). The histological parameters that were assessed following LPS-induced lung injury includes (**a**-**e**) the neutrophilic infiltration, edema and fibrosis. Similarly, the panels (**f**-**j**) includes the changes in the bronchial architecture following LPS-induced ALI. The H and E staining showed significant improvement in the histopathological parameters in the treatment control compared to the negative control. Similarly, the histological changes (**k**) were quantified using score of 0–4 and the continentalic acid improved the histological features. The MPO assay (**l**) performed to assess the level of the neutrophilic indicator in the lung tissue following establishing ALI. The continentalic acid showed marked reduction in the MPO level compared to the negative control. All data were expressed as mean (n = 8) ± SD. ^###^*p* < 0.001 compared to control group; ^*^p < 0.05, ^**^p < 0.01 and ^***^p < 0.001 compared to LPS-treated group

Normal group showed no histopathological changes as shown in Fig. 5. Clear histopathological changes were detected in superior right lobe of lung for LPS treated group. Histopathological score = 10 was observed for LPS treated group, which indicates severe histological damage as compared to histopathological score of vehicle control group = 0. Continentalic acid treatment 50 mg/kg and 100 mg/kg remarkably reduced neutrophilic infiltration and significantly improved lung histopathology in a dose dependent manner. Similarly, the histopathology study showed marked improvement in the tracheal architecture compared to the LPS treated group as shown in the Fig. 5.

### Effect of the continentalic acid on MPO activity

The MPO activity served as marker of neutrophilic infiltration into the site of the inflammation. The MPO concentration was markedly increased in the lung tissue following LPS administration [33]. The continentalic acid markedly reduced the activity of the MPO activity in the lung tissue compared to the negative control group. Similarly, the positive control group also reduced the level of the MPO concentration significantly as shown in the Fig. 5.

### Effect of continentalic acid on Nrf2 and iNOS expression using immunohistochemistry

**Fig. 6:**
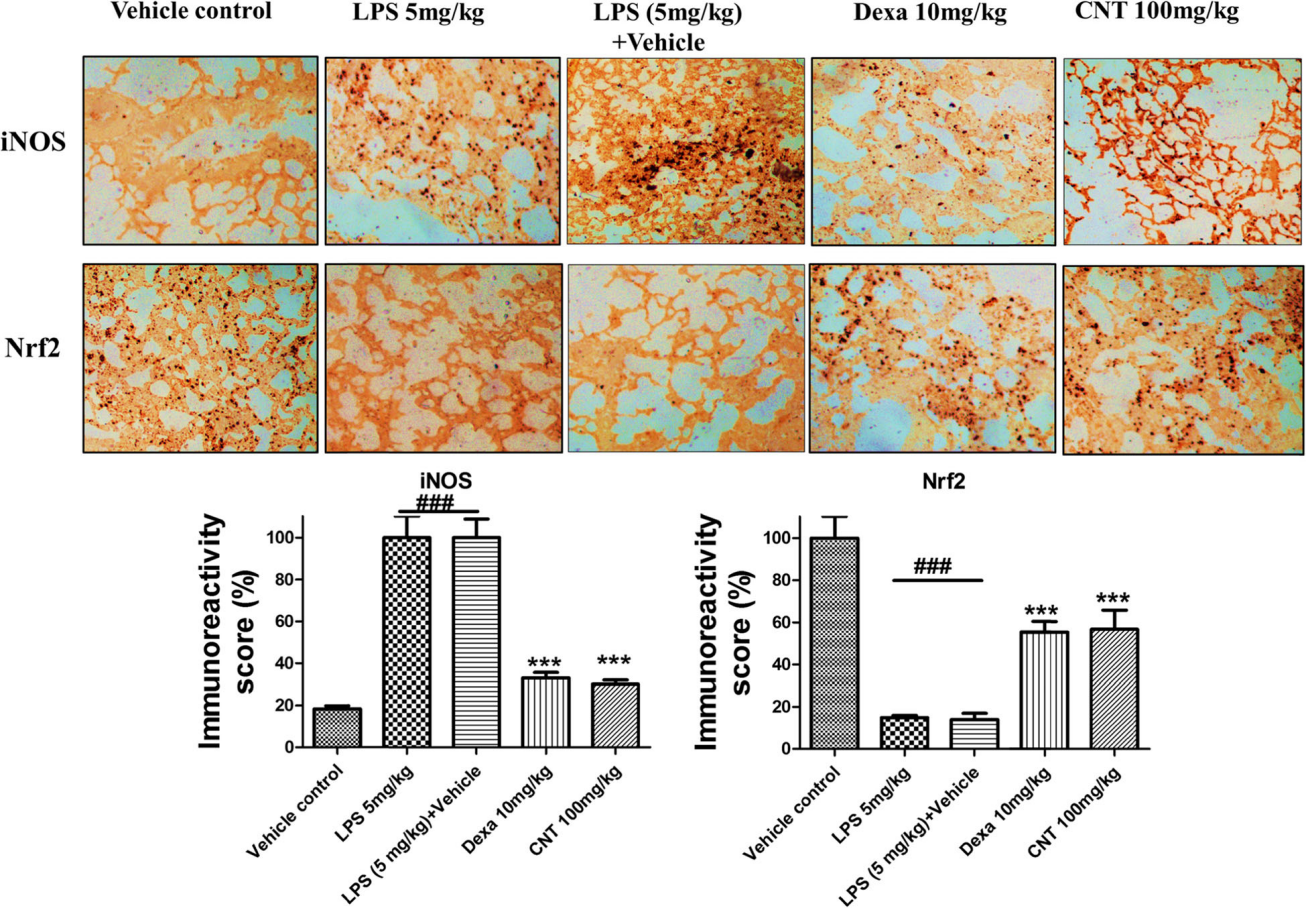
The effect of the continentalic acid on the iNOS and Nrf2 level using immunohistochemistry in the lung tissue following LPS-induced ALI. The changes in the iNOS and Nrf2 level were assessed in all the treated groups and expression were quantified. The continentalic acid markedly attenuated the expression level of iNOS protein compared to the LPS treated group. However, the continentalic acid enhanced the expression level of the Nrf2 protein significantly compared to the LPS treated group. All data were expressed as mean (n = 8) ± SD. ^**###**^*p* < 0.001 compared to control group; ^*^p < 0.05, ^**^p < 0.01 and ^***^p < 0.001 compared to LPS-treated group

The Nrf2 is the part of the cell endogenous antioxidant enzymes system and kept inactive within the cytosol by the Keap1 [41]. Once body encounter any oxidative stress, the Nrf2 get activated and translocated to the nucleus to influence the concerned genes [41]. The contenantalic acid markedly enhanced the Nrf2 level following LPS-induced lung injury compared to the negative control group. The level of the iNOS enhanced during inflammatory insults. The LPS administration markedly enhanced the iNOS level, however, the continantelic acid markedly decreased the iNOS level as evident from the Fig. 6.

### Docking results

**Fig. 7:**
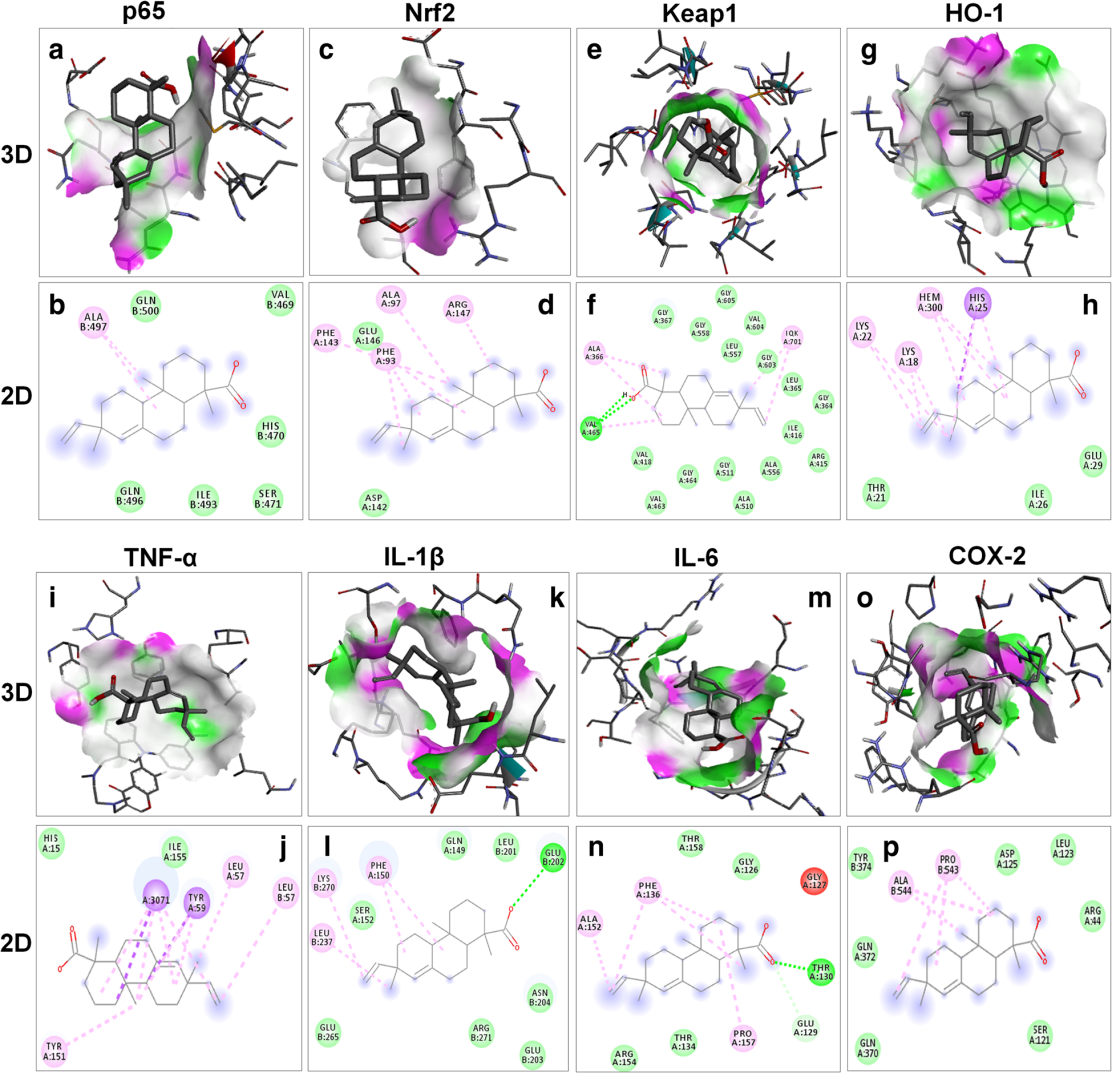
The binding interaction of the continentalic acid with various protein target such as p65 (PDB-ID: 1vkx), Nrf2 (PDB-ID: 2flu), Keap1 (PDB-ID: 4iqk), HO-1 (PDB-ID: 1ubb), TNF-α (PDB-ID: 2az5), IL-1β (PDB-ID: 1itb), IL-6 (PDB-ID: 1p9m) and COX-2 (PDB-ID: 5ikq) with their subsequent 3D and 2D structure. The docking analysis was done to calculate the binding energy, vander wall forces and type of binding. The interaction of the continentalic acid with the p65 (**a**, **b**), Nrf2 (**c**, **d**), Keap1 (**e**, **f**), HO-1 (**g**, **h**), TNF-α (**i**, **j**), IL-1β (**k**, **l**), IL-6 (**m**, **n**) and COX-2 (**o**, **p**) was shown in both 2D and 3D view. The 3D view show the binding pocket within the ligand orient, while the 2D images show the interactive amino acids interacting with the ligand and type of interaction

**Fig. 8:**
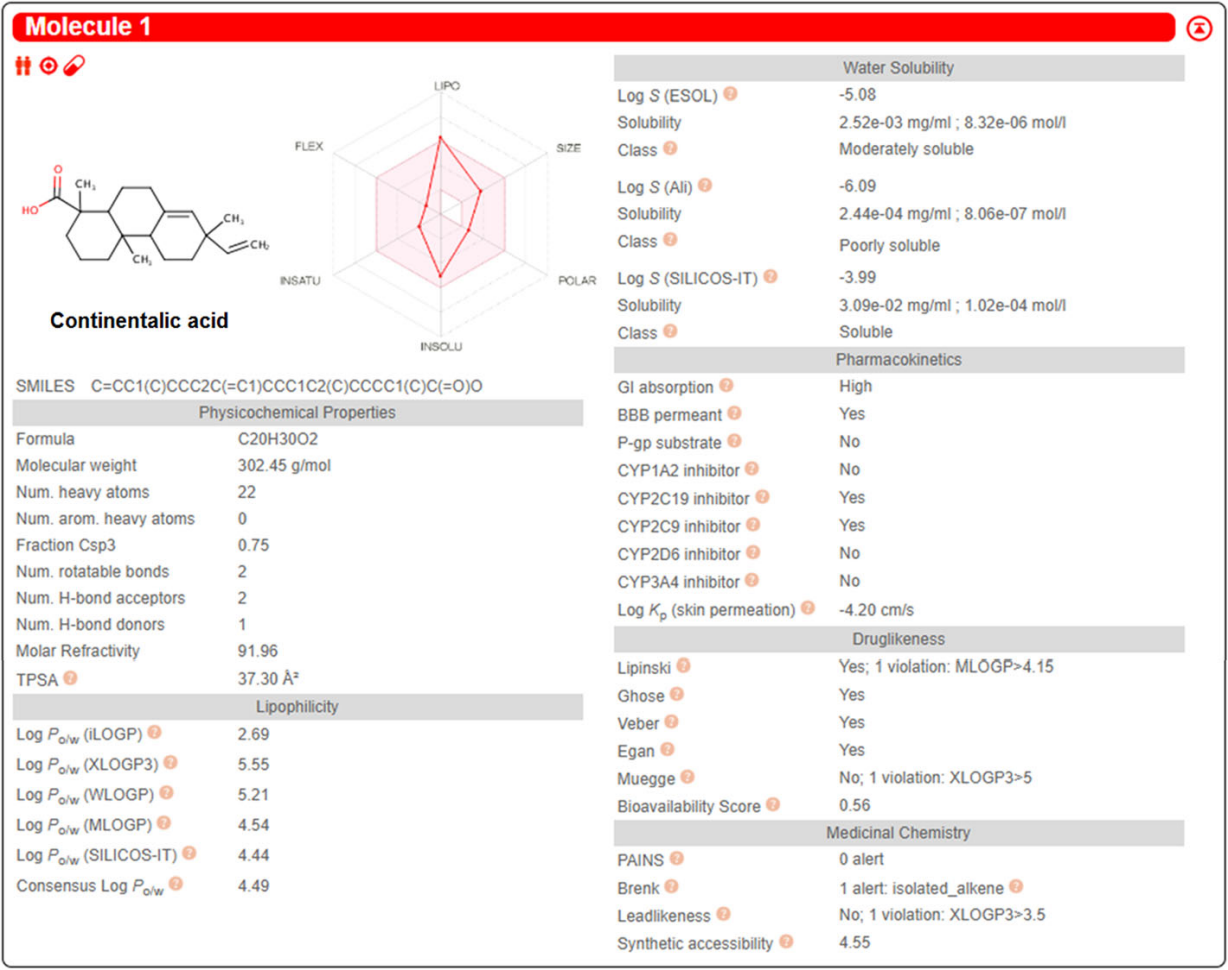
The assessment of the pharmacokinetic and physicochemical parameters of the continentalic acid using Swiss target prediction software. The various physicochemical parameters that were assessed includes bioavailability, absorption, distribution and water solubility. The continentalic acid followed the Lipinski, Ghose, Veber and Egan rule, while showed the bioavailability score of 0.56

**Fig. 9:**
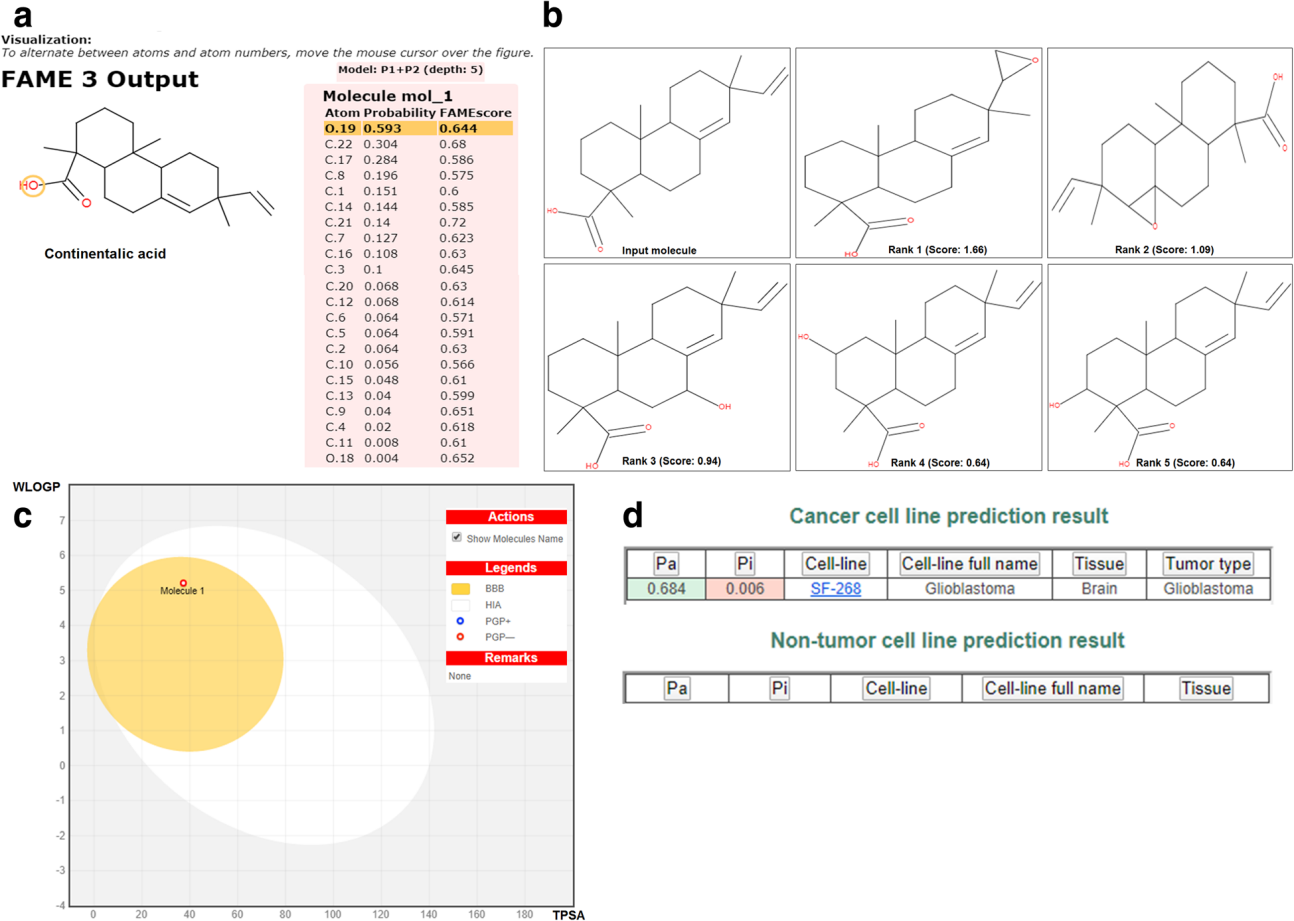
The assessment of FAME1 (**a**), possible metabolites (**b**), blood brain barrier permeability (**c**) and toxicity against the cell lines (**d**) using molecular docking software. The boiled egg analysis showed blood brain permeability of the continentalic acid, no toxicity against the normal cell lines and the product of the phase I metabolism

Molecular docking was used to investigate the affinity between continentalic acid and several protein targets implicated in the pathogenesis of lung injury. Ligand (continentalic acid) was docked against (Keap-1 PDB-ID: 4iqk), (Nrf2 PDB-ID: 2flu), (p 65 PDB-ID: 1vkx), (HO-1 PDB-ID: 1ubb), (TNF-a PDB-ID: 2az5), (IL-1Β PDB-ID: 1itb), (IL-6 PDB-ID: 1p9m), (COX-2 PDB-ID: 5ikq) and the results obtained in the form of binding affinity and bond length are shown in Table 2. The continentalic acid showed high binding affinity for the Nrf2, Keap1, HO-1, p65, TNF-α, IL-1β, IL-6 and COX-2 protein as shown from the Fig. 7. The pharmacokinetic analysis using Swiss target prediction software showed that continentalic acid exhibit moderate water solubility, high GIT absorption, BBB permeability, and interaction with the cytochrome p450 system. Furthermore, the continentalic acid followed most of the drug likeness rule including Lipinski rule. As for as the toxicity is concerned, the continentalic acid exhibited no toxicity against the various cell lines using CLC-Pred software. The various possible metabolites, their route and ranked according to their production were estimated as shown in the Figs. 8 and 9.

**Table 2:** Molecular docking of continentalic acid with various protein targets

Proteins	PDB ID	Amino Acid	Binding energies (kcal\mol)
**Keap 1**	4iqk	VAL465	−9.0
**Nrf2**	2flu	PHE93	−8.4
**P65**	1vkx	ALA497	−7.2
**HO-1**	1ubb	HIS25	−6.5
**TNF-α**	2az5	TYR59	−8.2
**IL-1β**	1itb	GLU202	−6.5
**IL-6**	1p9m	THR130	−6.5
**COX-2**	5iqk	PRO543	−7.5

## Discussion

Acute lung injury (ALI) is an inflammatory disease characterized by loss of the alveolar-capillary membrane integrity, formation of lung edema, thus, resulting in impairment of arterial oxygenation (Bhatia, 2004). The principal characteristics of ALI includes elevated levels of pro-inflammatory cytokines, depletion of antioxidant enzymes, compromised pulmonary vascular permeability, significant neutrophilic infiltration and impaired gaseous exchange [42].. Regardless of all novelties in emergency medicine, the mortality rate associated with ALI remains as high as up to 30–40% [43]. Therefore, the effects of continentalic acid on survival rate was evaluated. Post-treatment with continentalic acid significantly reduced LPS-induced death. The survival rate during 24 h in high dose of continentalic acid (100 mg/kg) treatment groups was significantly higher (80%) as compared to LPS groups (20%). The present results indicate that continentalic acid 100 mg/kg has the potential to reduce mortality rate associated with ALI.

LPS is a glycolipid that is present in the cell wall of Gram negative microbes which comprises of numerous disaccharide units and a polar lipid head group [44]. LPS ties to its exceptional LPS binding protein (LBP) that creates a LPS/LBP complex. This complex at that point triggers the activation of the CD14/TLR4 receptor on the monocytes and macrophages [44]. This complex is also involved in transduction of inflammatory signal resulting in the regulation of the inflammatory cytokines [45]. Inflammatory cytokines play an important role in the pathogenesis of ALI [10]. Among cytokines TNF-α is responsible for transduction and amplification of inflammatory response [46] . IL-1β causes deterioration of lungs epithelial and endothelial cellular layer [47]. IL-6 is involved in formation of lung edema and protein rich hyaline membrane [48]. Production of these cytokines play a critical role in the pathogenesis of ALI and are also involved in production of ROS [41, 49]. There for, inhibition of these cytokines could ameliorate acute lung injury. In this study LPS treated mice shown remarkable elevation of IL-1β, IL-6 and TNF-α level as compared to control group. However, continentalic acid treatment significantly inhibited production of pro-inflammatory cytokines, compared with LPS treated group. Similar results were observed with dexamethasone treatment. The results suggested that continentalic acid suppressed LPS-induced pro-inflammatory cytokine production by preventing NF-κB activation. In the present study, we have demonstrated that continentalic acid have ability to inhibit LPS-induced ALI.

Neutrophils also contribute in the pathogenesis and development of ALI [17]. Administration of LPS, either by an intravenous or intraperitoneal route leads to entanglement of neutrophils in pulmonary capillaries and compromised deformability [50].. The neutrophil entrapment and inflammatory response causes alteration in pulmonary capillary permeability which results in edema formation with protein rich hyaline membrane [51]. In the present study complete blood count results demonstrates that neutrophils concentration were quite higher in LPS-induced group as compared to normal and treatment with continentalic acid remarkably normalized the neutrophil level. In acute lung injury exaggerated stimulation of phagocytes and neutrophils causes an uncontrolled release of reactive oxygen species (ROS) [52]. Numerous studies reported that oxidative stress is implicated in the pathophysiology of lung injury [53]. In the present study LPS administration significantly dropped the level of endogenous antioxidants such as GSH, GST and Catalase respectively while increased MDA level. On the other hand, continentalic acid post-treatment remarkably increase antioxidant enzymes level such as GSH, GST and Catalase while decreased MDA level.

The histopathological analysis revealed that continentalic acid treatment reduced histopathological changes. Normal group showed no histopathological changes. Clear histopathological changes were detected in superior right lobe of lung for LPS treated group. Histopathological score = 10 was observed for LPS treated group, which indicates severe histological damage as compared to histopathological score of vehicle control group = 0. Continentalic acid treatment 50 mg/kg and 100 mg/kg remarkably reduced neutrophilic infiltration and significantly improved lung histopathology in a dose dependent manner.

Molecular docking was used to investigate the affinity between continentalic acid and various protein targets. Ligand (continentalic acid) was docked against (Keap-1 PDB-ID: 4iqk), (Nrf2 PDB-ID: 2flu), (p 65 PDB-ID: 1vkx), (HO-1 PDB-ID: 1ubb), (TNF-a PDB-ID: 2az5), (IL-1Β PDB-ID: 1itb), (IL-6 PDB-ID: 1p9m), (COX-2 PDB-ID: 5ikq) and the results obtained in the form of binding affinity and bond length. The lowest binding energy value represents highest binding affinity (Trott, 2010). The continentalic acid showed high binding affinity with various protein targets such as Nrf2, Keap1, p65, TNF-α and COX-2. The continentalic acid showed interaction with the protein tragte such as Nrf2, Keap1, p65, COX-2 and TNF-α more strongly than the other proteins such as IL-1β, IL-6 and HO-1, which indicates that protective activity of the continentalic acid might involve these antioxidant and anti-inflammatory proteins.

## Conclusion

In conclusion, the continentalic acid 100 mg/kg has the potential to reduce mortality rate associated with ALI. In present study, complete blood count results demonstrates that treatment with continentalic acid remarkably normalized the neutrophil level. In the present study continentalic acid remarkably increased level of antioxidant enzymes such as GSH, GST, Catalase and SOD, while decreased the MDA, MPO and NO level. Furthermore, the continentalic acid improved the histological parameters and attenuated the inflammatory cytokines. Furthermore, the docking analysis showed good interaction with the various protein targets. However, more in depth investigation is still required to explore the molecular mechanism, but the current results showed that continentalic acid might be a candidate for treatment of acute lung injury.

## Data Availability

The corresponding author will provide the date used in the current study upon request.

## Abbreviation

Nrf2: Nuclear factor erythroid 2-related factor 2
TNF-α: Tumor necrosis factor-α
COX-2: Cyclooxygenase-2
NF-κB: Nuclear factor kappa-light-chain-enhancer of activated B cells
GST: Glutathione-S-transferases
MPO: Myeloperoxidase
NO: Nitric oxide
SOD: Sulphur oxide dismutase
LPO: Lipid peroxidase
HO-1: Hemeoxygenase-1
MDA: Malonaldehde
IL-6: Interleukin 6
IL-1β: Interleukin-1β
Keap1: Kelch-like ECH-associated protein 1
iNOS: Inducible nitric oxide synthase
